# Caregivers’ perceptions of digital screen use by children from different strata of social vulnerability

**DOI:** 10.1590/0034-7167-2025-0075

**Published:** 2026-08-21

**Authors:** Anniely Rodrigues Soares, Iolanda Carlli da Silva Bezerra, Valquíria Francisca de Moura, Rafaella Karolina Bezerra Pedrosa, Gabriela Lisieux Lima Gomes, Altamira Pereira da Silva Reichert

**Affiliations:** I Universidade Federal da Paraíba. João Pessoa, Paraíba, Brazil

**Keywords:** Child Day Care Centers, Caregivers, Children, Screen Time, Social Vulnerability., Guarderías Infantiles, Cuidadores, Niños, Tiempo de Pantalla, Vulnerabilidad Social.

## Abstract

**Objectives:**

to understand caregivers’ perceptions of screen use by children from different strata of social vulnerability.

**Methods:**

a qualitative study was conducted with caregivers of preschool children in daycare centers in a municipality in northeastern Brazil between October 2023 and June 2024. The focus group technique and inductive thematic analysis were used, based on the dimensions of digital screen use, namely time, content, nature, and context.

**Results:**

children use screens beyond the recommended time, with varied content and often with little supervision, which can expose them to inappropriate content.

**Final Considerations:**

it is essential to guide caregivers on the impacts of screen use on child development and the importance of adequate monitoring.

## RESUMEN

**Objetivos:**

comprender las percepciones de los cuidadores sobre el uso de pantallas por parte de niños de diferentes estratos de vulnerabilidad social.

**Métodos:**

se realizó un estudio cualitativo con cuidadores de niños en edad preescolar en guarderías de un municipio del noreste de Brasil entre octubre de 2023 y junio de 2024. Se utilizó la técnica de grupo focal y análisis temático inductivo, con base en las dimensiones de uso de pantallas digitales: tiempo, contenido, naturaleza y contexto.

**Resultados:**

los niños usan pantallas más allá del tiempo recomendado, con contenido variado y, a menudo, con poca supervisión, lo que puede exponerlos a contenido inapropiado.

**Consideraciones Finales:**

es esencial orientar a los cuidadores sobre los impactos del uso de pantallas en el desarrollo infantil y la importancia de un monitoreo adecuado.

## INTRODUCTION

Child development is marked by regulated normative influences, with a strong socio-historical influence, in which significant events define the behavior and attitudes of a generation, such as the categorical presence of technological innovations^(1)^.

With the advent of modern technology, smartphones, tablets, computers, and digital toys have become incorporated into children’s routines, who are often preoccupied with digital content. The excessive use of these technologies results in many challenges for child health, development, and behavior^(2,3)^.

A study identified that 63.3% of children (n=180) between 24 and 42 months of age are exposed to digital screen time equal to or greater than two hours/day^(4)^, contradicting Brazilian and international recommendations for screen time of up to one hour per day in the 2 to 5 year age group^(5,6)^.

The time and manner in which digital screens are used are related to the benefits and/or drawbacks of this practice in children’s lives. Among the positive influences are improved learning skills, easy access to video calls with family members, enhanced creativity, and self-expression. On the other hand, negative influences include technology addiction, increased stress levels, reduced physical activity, language delays, sleep deprivation, emotional distress, and behavioral and relationship problems^(3)^.

Among the children exposed to the harmful effects of digital screens due to excessive screen time are those in situations of social vulnerability. A study that assessed 13,891 European children in socially vulnerable situations, characterized by the absence of a parental social support network, non-traditional family composition, migration status, and/or unemployment of one or both parents, identified a higher risk of them exhibiting excessive screen time and being less active in sports groups, compared to non-vulnerable groups^(7)^.

In Brazil, data indicates that 32 million children and adolescents are in situations of vulnerability and deprivation. Research led by the United Nations Children’s Fund identified that, among the eight indicators that make up multidimensional poverty, three showed a significant worsening between 2020 and 2022, namely food, education, and income. On the other hand, access to information, i.e., to TV and the internet at home, showed a significant improvement up to 2020, but it is still very unequal^(8)^.

The concept of social vulnerability can be applied to people experiencing adverse situations, being associated with risk factors that negatively affect individuals and their daily lives. These risk factors, such as excessive screen time by children, can be conditions or behaviors that cause negative or undesirable effects and increase the likelihood of adverse consequences for psychosocial development, potentially compromising individuals’ health and well-being^(9)^.

Parents play a crucial role in organizing their children’s screen time, as children, due to their stage of life, are more vulnerable to the dangers of digital screens. Extra settings and passwords on electronic devices, as well as removing digital screens from bedrooms, establishing time limits, and monitoring their own screen time are viable strategies that parents can adopt to mitigate excessive screen use by children^(10)^.

Despite the importance of parents, their knowledge about recommended screen time and methods of digital screen use for children, as well as its benefits and drawbacks, is scarce. Research conducted in the United States, entitled “Common Sense Media”, showed that approximately 60% of parents of children aged 0 to 8 years predominantly believe that their children use the right amount of screen time, and most emphasize that they are not concerned about the impact of screens on children or the quality of the content available to them^(11)^.

Understanding by caregivers, educators, and healthcare professionals about the potential risks of excessive screen use is fundamental to promoting healthy child development. This understanding leads to greater emphasis on other activities that foster cognitive, linguistic, and socio-emotional skills, which are then incorporated into children’s routines^(10)^.

Thus, early and appropriate educational interventions aimed at the caregiver group constitute a public health demand and a commitment to overall child development. In this health context, nurses are important agents in promoting dialogues that include stimuli for child development, reduction of passive activities, and control of the content and time spent on screens^(12)^. This is crucial, given that the family environment is the primary place where children use digital technologies. Parental mediation regarding the use of digital technology and screen time should be part of the interactions between parents/caregivers and children. However, permissiveness regarding excessive access to and use of screens is mainly caused by the parental need to keep children occupied^(13)^. Based on the literature, the following question emerges: what are the perceptions of caregivers of preschool children from different strata of social vulnerability regarding their children’s use of screens?

## OBJECTIVES

To understand caregivers’ perceptions of digital screen use by children from different strata of social vulnerability.

## METHODS

### Ethical aspects

This study corresponds to the third phase of a larger project entitled “*Promoção da saúde de pré-escolares em vulnerabilidade social: um estudo de intervenção em creches de João Pessoa-PB*”, approved by the Research Ethics Committee of a public higher education institution in Paraíba.

The research adhered to the ethical guidelines of Resolution 466/2012 of the Brazilian National Health Council. Participants were informed about the voluntary nature of their participation in the study, the associated risks, and the need to sign the Informed Consent Form. Anonymity was maintained using the abbreviation “FG” (FG1, FG2... FG8), which corresponds to the chronological order in which the focus groups (FGs) were conducted, combined with the quality chosen by the caregiver in the initial dynamics of the FG and the vulnerability stratum, such as “FG5-Courage, I”.

### Theoretical-methodological framework

This study was developed from the perspective of the digital screen use dimensions proposed by Fitzpatrick *et al*., which are screen time, content, nature, and context. Therefore, data were analyzed based on screen time, which corresponds to screen use duration; content, whether they are educational videos and applications, the speed of the action presented in the videos, and whether they are age-appropriate, pro-social, or violent; nature, whether their use is passive or active; and context, which represents the moment, the location/physical environment, and caregivers’ justifications for using screens with their children^(14)^.

### Study design

This is a qualitative, descriptive, and exploratory study, using the Consolidated Criteria for Reporting Qualitative Research as a guide to inform the reporting of the research process.

### Study setting

The research took place in eight Municipal Early Childhood Education Centers (In Portuguese, *Centros Municipais de Educação Infantil* - CMEIs), also known as daycare centers, in João Pessoa, Paraíba, Brazil. In two daycare centers, it was not possible to conduct the FGs due to violence in the community and closure for renovations.

In 2022, the municipality had 93 CMEIs, which were classified into five strata of social vulnerability, depending on the neighborhood in which they were located (I - very low vulnerability; II - low vulnerability; III - medium vulnerability; IV - high vulnerability; V - very high vulnerability) and the income, infrastructure, education, gender, work, and family composition variables, according to the municipality’s social topography^(15)^.

These variables are established in the Basic Operational Standard/Brazilian Unified Health System, which presents the methodological bases for measuring the presence of social vulnerabilities in a given territory so that the presence of a single characteristic mentioned above already indicates social vulnerability to some degree, namely: 1) income (family *per capita* income less than ¼ of the minimum wage (MW); family *per capita* income up to ½ MW); 2) infrastructure (inadequate housing; households with two to three residents per bedroom (3:1 density)); 3) education (4- to 14-year-olds not attending school; heads of household with less than four years of education); 4) gender (female heads of household without a spouse); 5) employment (unemployed person over 25 years old with four years of education; working person aged 10 to 15); 6) family composition (presence of a person over 60 years old with *per capita* family income less than ½ minimum wage; presence of a person with a disability and *per capita* family income less than ½ minimum wage; number of children up to 14 years old with *per capita* family income up to ½ minimum wage and a head of household with less than four years of education; number of children under 15 years old; and female head of household without a spouse)^(15)^. It is worth highlighting that this distribution is consistent with the social topography of the municipality, available in 2022.

In this context, the selection of daycare centers was carried out through proportional stratified sampling, adopting a random procedure based on data provided by the Municipal Department of Education.

### Methodological procedures

The educational intervention on the use of digital screens was developed with caregivers of preschool children regularly enrolled in daycare centers, through group discussions, videos, and interactive activities (second phase of the project). At the end of the educational intervention in each daycare center, the date for the next phase of the study (FGs) was agreed upon with the group of caregivers and management, according to caregivers’ availability and the daycare center’s schedule. A reminder was sent in advance via messaging app the week of the meeting.

### Study participants

The purposive sampling technique was used, inviting all caregivers of preschool children duly enrolled in daycare centers and aged 2 to under 5 years old, who had previously participated in the educational intervention, to participate in the study. Caregivers who arrived late after the meeting discussion (FG) were not included in the study.

### Data collection and organization

The third phase of the project took place between October 2023 and June 2024, between one and three months after the educational intervention to promote child health, in order to mitigate memory bias.

The FG was the data collection technique implemented, which aims to express opinions that promote an understanding of participants’ behaviors and perceptions^(16)^.

The FG was conducted in eight daycare centers, with one FG carried out in each center. It was led by a moderator, the principal investigator, supported by an observer, a faculty member from a higher education institution with experience in FGs, and by assistants (undergraduate and graduate students) from a child and adolescent health study group at a public university.

To begin the FG, a “qualities dynamic” was conducted, which consisted of reading a list of qualities, and each participant chose the one that best represented them. The chosen quality was written on participants’ name tag, becoming their codename “FG”.

Immediately afterwards, a surprise box containing loose words was presented, and participants were invited to form a sentence that addressed the FG’s trigger question: “How does your child use digital screens?”.

The FG discussions were audio-recorded and lasted an average of 60 minutes. The closing criterion adopted was saturation, when a moderator determines that the researched phenomenon can be understood from the information already collected^(17)^. The data *corpus* was transcribed in full, but participants were not validated.

### Data analysis

Empirical data were analyzed using inductive thematic analysis, a flexible and recursive technique composed of the following phases: familiarization with the data, discourse transcription, and exhaustive reading of the *corpus*; initial code development - coding of important and most recurrent aspects and grouping of data extracts related to each code; search for topics - joining of extracts of related codes into potential topics; review of topics - thematic map development with its subtopics and codes and verification of all selected extracts; topic definition and naming - adjustment of the titles of topics, subtopics, and codes; final text production - final analysis of the selected extracts and their relationship to the research question and objective^(18)^.

Data *corpus* analysis was validated by two researchers with experience in qualitative research (peer review). The principal researcher previously performed the analysis, validating it, and subsequently, the material was sent for validation by the umbrella project coordinator, in addition to being analyzed by the research group.

All data *corpus* analysis followed national recommendations on the use of digital screens by children, considering a usage time of up to one hour for children aged 2 to 5^(5)^; above that, the time was considered excessive.

To maintain the anonymity of FG participants, the abbreviations FG1, FG2 [...] FG8 were adopted, which consist of the chronological order in which the FGs were carried out, plus the quality chosen by them in the initial dynamics of the FG (respect, hope, love, among others) and the social vulnerability stratum of the daycare center (I, II, III, IV and V), resulting, for instance, in the code “FG1-Gratitude, III” [...].

## RESULTS

Eight FGs were conducted in daycare centers serving social vulnerability strata I, II, III, and IV, with two daycare centers from each stratum. It was not possible to conduct FGs in daycare centers serving stratum V due to community violence and building renovations.

Considering the different social realities encountered, seven FGs were conducted in person and one remotely, which facilitated data collection with greater caregiver participation. Thus, 30 caregivers participated in this stage, with an average of four participants in each FG. The majority were mothers of children, declared as primary caregivers, aged between 18 and 30 years, single, with 10 to 12 years of education, and with some form of paid employment. Children were mostly between 36 and 47 months old, female, and brown.

Textual *corpus* analysis, which was based on dimensions related to screen use, resulted in the construction of the topic “Constituent elements of screen use by children in social vulnerability” and the sub-topics “Digital screen time”, “Contexts of digital screen use”, “Nature of digital screen use”, “Content consumed on digital screens”, and “Experiences of preschoolers’ families with digital screens”.

### Topic: Constituent elements of screen use by children in social vulnerability

#### Subtopic I: Digital screen time

Digital screens are embedded in the various contextual systems of human development. Considering all aspects, such as time, context, content, and the nature of their use by preschoolers, allows for a broader understanding of a phenomenon with a significant impact on early childhood.

The amount of time children spend on digital screens is a concern, as all caregivers reported their children using screens predominantly for around two to three hours a day, a time considered excessive for their age group.

*Look, when she’s here with me, she stays for about three hours, then I turn it off. For instance, in the morning, she wakes up and goes to play. Then, I leave her watching TV for about three hours, because now the tablet is broken, so it’s just TV.* (FG5-Courage, I)

*If I let her, she’ll spend about five hours just looking at the screen. If the phone doesn’t die, if I don’t take it away, she’ll stay like that.* (FG8-Respect, III)

*At home, when he gets home from school, he asks for screen time, and I let him have it for about two hours. That’s the time I have to do my things. He stays from 6:00 p.m. to 8:00 p.m. Then, around 7:30 p.m., I start turning it off* [...]. *I turn it off, and he doesn’t ask for it anymore, he doesn’t watch it anymore. And in the morning, it’s during that interval, that half-hour, that he watches.* (FG3-Love, IV)

#### Subtopic II: Contexts of digital screen use

The contexts for using digital screens are diverse. Children are in daycare during the morning and afternoon shifts, which protects them from excessive screen exposure at home. However, all their time outside of daycare is permeated by screen use, often so that their caregiver can perform domestic or personal tasks, or even during the commute from home to daycare.

*No, I would put him there* [in front of the television]. *He would ask to watch, and I would let him. I would go wash my dishes, make lunch. I know it’s wrong for me, but I had no choice. I let him because I could tidy things up, sweep the house.* (FG1-Gratitude, III)

*The thing is, I have very little time with them because they’re in daycare full-time, so my time with them is mostly at night. Then I have my course, my internship, so my time with them is very limited. So, but* [...] *unfortunately, the screens* [...]. (FG3-Respect, IV)

*At my house, screen time isn’t going very well. Even to come* [to daycare]*, she has to bring her cell phone, otherwise she cries all the way here. She’s gotten used to watching her cartoons.* (FG3-Patience, IV)

*In the morning, he also has this habit. When we’re coming* [to daycare] *in the car, he always asks for my cell phone, and then I end up giving it to him, you know?* (FG3-Love, IV)

In the context of public spaces, such as restaurants and churches, screens have become a “pseudo-ally” for caregivers to keep children quiet and still. However, the positive decision of two caregivers not to offer screens as “electronic babysitters” in public spaces should be highlighted.

*I’m not going to lie, when we go out, I want him to be quiet. I say, “Go on, Mommy, come here, look here”. I’m not going to lie, I do it. I know it’s wrong, but I do it to keep him quiet, but I’m going to try to control myself so I don’t do it anymore, because it’s hurting him.* (FG1-Gratitude, III)

*And really, I get judged and pointed at a lot, you know? And the boy can’t even cry without it being annoying, especially in a restaurant. Because you see the children sitting there, all quiet, but they’re all eating, without even knowing the color of the food* [while using screens]*. And my son is different, you know? He eats knowing what he’s eating. He orders what he wants and he eats what he wants, knowing what he’s eating. He’s not hypnotized by a screen, you know? But then we get judged.* (FG5-Resilience, I)

*I went to mass with him. He sees the other children, and their parents give them* [the digital screen] *so he can watch the mass. Then he came to get mine. I said, “No, you can’t. This is church, you can’t use cell phones”. Then he comes, goes, puts it in my bag and accepts it* […] *he’s very accepting.* (FG8-Victorious, III)

#### Subtopic III: Nature of digital screen use

Caregivers play an important role in choosing the contexts in which their children will use screens, as well as whether the use will be passive or active (natural). The results indicated passivity among children in relation to screens, even though some caregivers are aware of the importance of their presence in ensuring that screens are a beneficial tool for children.

*It’s a tool, it’s a double-edged sword, isn’t it? Because, to be a productive tool, it has to be supervised by an adult. And, often, it’s not possible to supervise. That’s the big problem with screens, because there’s so much going on. My son doesn’t watch much* [...] *he watches short films, but not a lot of nonsense. He watches animal videos; there are many cool things and experimental videos. But, for that to work, it has to be done with the parents, and it’s never possible to use them that way.* (FG3-Gratitude, IV)

*I’m always very close. If I’m not sitting with him, if I’m not with him in the hammock or on the floor, in some corner, I’m spending all my time with him. Because, to break the monotony, you know? Because the boy gets so engrossed in it, so I give him a little break.* (FG5-Resilience, I)

The occasional presence of caregivers during children’s screen time was primarily intended to assess and modify the content they were already consuming.

*When I see that something is heavy, and I hear it, I go there and say, “No, you can’t do that”, and that’s when he throws a tantrum, goes outside, and throws himself on the floor.* (FG1-Courage, III)

*In my case, they’re alone, but then I regulate what they’re watching, of course. When I hear those worldly songs, I tell them to change it, obviously* [...] *when I see it, I say, “Change that, girl”, and she changes it. I can hear it from the room. I sometimes check on them to regulate, but I’m not around 24 hours a day, but I do regulate. Which is the right thing to do, isn’t it? Even if you’re not physically close, you have to be there, watching what they’re doing.* (FG4-Love, II)

#### Subtopic IV: Content consumed on digital screens

The content children watched was diverse, including videos available on digital platforms (YouTube^®^, Kwai^®^, and TikTok^®^), music, cartoons, and movies. Since screen time was not systematically monitored by caregivers, it is unknown whether the content adhered to the age rating for digital content suitable for children.

*She calls it a drawing, but it’s not. There are some* [videos] *that I watch and some that she puts on her phone herself, on YouTube^®^. When I see them, I edit them in my own way, but without me seeing, she does it. And she’s 3 years old, smarter than me.* (FG3-Patience, IV)

*Because they watch Kwai^®^, which doesn’t have much good content and teaches some things that are no good.* (FG4-Love, II)

*She used TikTok^®^ a lot, which has a lot of heavy content, so I took it away from her.* (FG2-Patience, II)

*When he asks for more cartoons, because he doesn’t watch them often, I let him watch them, you know?* [...] *sometimes, I don’t put on the cartoon, I just put on the music for him to play with, just listening.* (FG5-Resilience, I)

*Sometimes I let my* [child] *stay a little longer, but sometimes she asks to watch a movie with me. Then I switch, because she’s watching something. I put on a children’s movie, then I put on a slightly more adult movie, but it doesn’t have any more of those kinds of scenes.* (FG4-Gratitude, II)

Regarding the type of digital screen, caregivers identified television as the preferred choice for home use, considering it less harmful to the child compared to cell phones and tablets.

*I don’t give her* [a tablet]*, the most she watches normally is on television, just regular programs. I had bought a tablet for her and her sister, but since I saw that the siblings were overdoing it, I took her sister’s tablet away too. So, the tablet has been sitting in the closet for three months, without either of them using it, because I’ve been so stressed out with teenagers that I’m just waiting for them to get on board the same way.* (FG1-Fortress, III)

*I don’t give her my cell phone. I let her watch more TV on YouTube^®^* [...] *well, now it’s just the TV because her tablet broke. I rarely give her a cell phone, so it’s just the TV.* (FG5- Courage, I)

*He doesn’t get a cell phone signal because we don’t let him. His grandmother lets him watch TV. He doesn’t want to play or anything. He even tells us to change the channel on the television.* (FG4-Patience, II)

#### Subtopic V: Experiences of preschoolers’ families with digital screens

Understanding how family members use digital screens is crucial, as it directly impacts the preschoolers’ relationship with screens. According to study participants, the use of digital media, in terms of time, context, content, or form, is also common among family members, freely and without restrictions.

*At home, it’s practically 24 hours a day. There’s television, the oldest one’s on his tablet, playing games. And, on TV, what I like to watch most at home is soap operas. I’m watching a soap opera, and then everyone else is watching. My oldest son, at least, likes that soap opera; it’s just me, him, and his dad. The youngest* [the child enrolled in daycare] *is on his cell phone. He uses YouTube^®^ a lot, which I’m going to try to take away from him.* (FG1-Courage, III)

*My daughter cries so much if I take her* [cell phone]*. She doesn’t stop for a minute, only if I give her the phone. I don’t know what to do. Then it’s a headache* [...] *if I don’t give it to her, she fights. At home, there are four children who fight over the phone.* (FG8-Respect, III)

When discussing the excessive use of digital screens within the family unit, some caregivers expressed feelings of guilt and a desire to change this practice.

*I feel very guilty because I’m on my phone a lot too. I blame my husband, and he blames me too, because we spend a lot of time* [on screens] [...] *I’m trying to control myself, both my husband and I, because we know that his behavior is also our fault. It distresses me a lot, so I’m trying to control myself so I don’t spend as much time on it as I used to.* (FG1-Gratitude, III)

*I have to do my best not to use my cell phone too much.* (FG1-Fortress, III)

## DISCUSSION

Based on caregivers’ perceptions, it was understood that digital screens are deeply embedded in the microsystem of children and their families from different strata of social vulnerability. They use them for excessive periods of time, in diverse contexts and with various content, in a passive manner. The practice of unrestricted use of electronics within the family unit was revealed, resulting in feelings of guilt among caregivers, but also a desire for change.

The use of screens has become a reality in children’s homes in Brazil and around the world, raising concerns about its impact on children’s neuropsychomotor development^(19)^. The stimuli offered in early childhood are essential for child development. Therefore, sensory interactions (touch, smell, proprioceptive stimuli) should come from familiar and environmental representations to promote positive conditions for child development, not from fictitious representations^(20)^.

Data reveals that preschoolers spend around two to three hours a day on screens, according to caregivers, which is considered excessive when considering available recommendations on the subject^(5,6)^. This is not an isolated finding, as a study conducted with 180 children, aged 24 to 42 months, enrolled in daycare centers shows that 63.3% reported screen time exceeding two hours per day^(4)^, which can have an impact on child development.

Regarding language development, for instance, a study conducted with children aged 1 to 3 years, which aimed to investigate the influence of the quantity, content, and context of digital media, revealed negative consequences related to screen time on the vocabulary of children aged 12 to 16 months. Moreover, children aged 17 to 36 months also showed negative effects amplified by longer exposure to digital media^(21)^.

Recent studies indicate that the impact of screen use on child health and development varies significantly depending on the content, exposure time, and social interaction involved. Research with Chinese children aged 3 to 6 identified that programs not specifically designed for children increase the risk of mental health problems^(22)^. Another study shows that passive screen time (such as passively watching television) is a predictor of lower quality of life, particularly in terms of physical well-being and social acceptance^(23)^.

According to study participants, children use digital screens in diverse contexts, both in public and private spaces, mainly in home environments. Caregivers indicated that digital tools were used to keep children quiet while they performed other domestic or other activities in collective spaces. However, some caregivers had a different understanding of screen use in public spaces and, when they allowed children to be children-i.e., when they did not have access to screens but to play with other instruments or toys-they felt judged by others. Furthermore, a Brazilian study that investigated the relationship between screen time in children up to 48 months of age and their caregivers showed a significant association between daily screen time and a higher prevalence of maternal symptoms of anxiety and depression^(24)^.

Research also shows that digital screens, whether in the home, other private environments, or public spaces, are widely used as “digital babysitters” to assist with parental needs and keep children entertained, quiet, and occupied while parents can enjoy moments of rest or perform household chores without interruption from the children^(12,25,26)^. However, within the family environment, screen time can stem from parents’ limited understanding of the impacts of screen use on early childhood social development^(12)^.

Low educational attainment among caregivers and/or limited access to health information are relevant factors to consider in screen use by vulnerable children, as social vulnerability is not only associated with low income and financial instability, but also with weaknesses in different aspects of life, affective and relational bonds, and unequal access to public goods and services^(27)^.

A study conducted in Switzerland with adolescents defined social vulnerability based on family socioeconomic status, relationship with parents, and academic performance, classifying them as non-vulnerable, moderately vulnerable, and highly vulnerable. The moderately and highly vulnerable adolescent groups showed a higher risk of smartphone or internet addiction, and the prevalence of excessive screen time increased with the degree of vulnerability^(28)^.

Guidance for parents regarding digital screens is effective in promoting a change in children’s screen time behaviors. An Indian randomized clinical trial, involving caregivers of children aged 2 to 5 years, focused on parental literacy regarding screen time and the home media environment, finding a significant reduction in average screen time in the post-intervention assessment in the intervention group, compared to the control group^(29)^.

Another factor that positively influences children’s screen time is their regular attendance at daycare centers. A North American study with 235 parent-child dyads under 2 years old found that those who attended daycare watched 43 minutes less screen time than those who did not^(30)^.

When screens are being used, it is crucial that they are viewed together by children and their caregivers, as this is a protective practice that promotes interaction and dialogue, reducing the negative impacts of technology on child development^(31)^ and the problems related to child health and safety in the online environment^(32)^. However, it was identified that children are sometimes unaccompanied, which allows them to consume digital platforms and content inappropriate for children, according to age ratings.

Children lack the maturity and discernment to handle the content presented on the internet, putting their safety, privacy, and social protection at risk. Consequently, they are subjected to the risk of becoming targets of online violence and abuse, such as nudes, sexting (sending erotic content), virtual rape, grooming (online enticement), pornography networks, pedophilia networks, hoaxes, dangerous challenges, phishing (obtaining information illegally), deepfakes (altering videos or photos with the help of artificial intelligence), cyberstalking (digital harassment that involves electronic means to stalk the victim), and cyberbullying (aggressive practice of intimidation and harassment in the virtual environment)^(32)^.

Given such serious problems, monitoring the content consumed by children should not be a one-off event aimed solely at changing the content. From children’s songs and cartoons to digital platforms like Kwai^®^ and TikTok^®^, there is a world of possibilities that are detrimental to child health. Studies have confirmed that videos on media platforms such as TikTok^®^ and Instagram^®^, and cartoons are the main types of content consumed by children, but that most of it is inappropriate for their age^(33)^, according to the Rating System^(34)^.

Concerning children’s use of television, caregivers consider it better for children to use than interactive media such as smartphones, tablets, and video games; therefore, they prioritize television use for their children.

A Brazilian study with 180 children aged 24 to 42 months found that, among the 94.5% of children exposed to screens, 61% used television, 41% smartphones, and 22% tablets. Therefore, television is still the main source of children’s screen exposure^(4)^. In contrast, another study with 104 children showed that 88.5% (n=92) use smartphones to consume digital content, demonstrating that this also represents a significant means of media exposure^(34)^.

Regardless of the screen type, its use will be largely influenced by parental habits^(35)^, referred to as parental technoference. With the increased use of smartphones in daily routines, technoference (the interference of technology in relationships) emerges as a new threat to family relationships and child development. A study conducted with 826 parents identified that 50% of children sometimes used smartphones when interacting with peers, and 41% when interacting with their parents. However, the use of smartphones during activities between parents and children/within the family, during meals or family trips, and during children’s personal activities such as studying and sleeping, is a practice that has generated dissatisfaction among parents^(36)^.

In the present study, caregivers acknowledged excessive screen time by children and the entire family. Feeling guilty about this reality, they expressed a desire to change this practice. It is noteworthy that the awakening to change behavior regarding screen use may stem from guidance received in educational intervention. However, feelings of guilt may have limited their accounts. In this context, research that assessed the impact of parental education focused on limiting screen time in early childhood demonstrated that, after intervention through an educational group, children showed a significant change in screen time, with a positive impact on their developmental skills and behavior^(37)^.

Excessive screen time prevents children from experiencing the interactions of home environments^(38)^. Including them in household activities, with their adapted participation in household chores, for instance, will help reduce the use of electronic devices, strengthening the bonds and interactions between parents and children.

Therefore, it is worth highlighting that health education for caregivers, especially those in situations of high social vulnerability, is valuable because it allows access to guidance on the ways and times children should use screens, as well as information on the impacts of excessive screen time. It can also suggest activities that promote healthy growth and development away from digital screens.

Considering that caregivers’ knowledge about the use of digital screens in early childhood is not equal, it was found that many caregivers were unaware of recommendations regarding screen time for children, as well as its repercussions on child health. Therefore, a positive influence was evident after the educational activity, demonstrated by the mothers’ understanding of the impact of screen use and their desire for changes in children’s screen time environment.

Therefore, the excessive, passive, and unrestricted use of screens by children was revealed across different strata of social vulnerability, a fact corroborated by other studies that confirm this unfavorable outcome^(20-22)^. Furthermore, some children may suffer greater disadvantages due to existing social inequalities within groups in situations of greater social vulnerability^(7)^, reinforcing the need for attention to the topic with a focus on health education.

### Study limitations

Among the limitations are the exclusion of preschool teachers - whose importance in childcare and in educating caregivers about the use of digital screens is undeniable - and the social stratification adopted, since, despite being official municipal data, they do not classify family vulnerability, but rather the region in which the preschool is located.

### Contributions to nursing and health

By presenting results that capture the context, nature, and content of screens, the study offers an innovative and in-depth look at digital screens and their use by children in socially vulnerable situations. This context is extremely relevant to health and nursing care, given the need to guide parents and society about the effects of screens on child development and the population’s health as a whole.

## FINAL CONSIDERATIONS

Caregivers of children from different socially vulnerable backgrounds perceived that their children’s screen time was sometimes excessive and in various contexts, with the aim of occupying children while they carried out their activities. This results in passive screen use by children, without adequate supervision and limited to sporadic assessment of the content consumed by children. Furthermore, television use is prioritized in the home, as it is perceived as a less harmful screen for children.

Therefore, it is suggested that caregivers’ access to information about digital screens and their impact on child development be expanded, especially for those in situations of greater social vulnerability, as well as information about recommendations from competent authorities. Caregivers’ positive perception regarding screens in child development is still a reality that can contribute to excessive and early exposure of children to digital screens, which, in the long term, could result in a generation dependent on technology and impact different areas of child development.

## Data Availability

The research data are available within the article.
