# Supplementary material for: Caregivers’ perceptions of digital screen use by children from different strata of social vulnerability
Source: Rev Bras Enferm. 2026 Aug 21;79:e20250075. doi: 10.1590/0034-7167-2025-0075 (PMC13505567; doi:10.1590/0034-7167-2025-0075)
Supplement: Translation

# Translation: Percepções de cuidadores sobre uso de telas digitais por crianças de diferentes estratos de vulnerabilidade social

**DOI:** 10.1590/0034-7167-2025-0075pt

**Authors:** Anniely Rodrigues Soares, Iolanda Carlli da Silva Bezerra, Valquíria Francisca de Moura, Rafaella Karolina Bezerra Pedrosa, Gabriela Lisieux Lima Gomes, Altamira Pereira da Silva Reichert

**Affiliations:** I Universidade Federal da Paraíba. João Pessoa, Paraíba, Brasil

**Keywords:** Creches, Cuidadores, Crianças, Tempo de Tela, Vulnerabilidade Social.

## Abstract

**Objetivos:**

compreender as percepções de cuidadores sobre o uso de telas por crianças de diferentes estratos de vulnerabilidade social.

**Métodos:**

estudo qualitativo, realizado com cuidadores de pré-escolares de creches de um município do Nordeste brasileiro entre outubro de 2023 e junho de 2024. Utilizaram-se a técnica de grupo focal e a análise temática indutiva, baseadas nas dimensões do uso de telas digitais, nomeadamente tempo, conteúdo, natureza e contexto.

**Resultados:**

as crianças usam as telas além do tempo recomendado, com conteúdos variados e, muitas vezes, com pouca supervisão, o que pode expô-las a conteúdos impróprios.

**Considerações Finais:**

é essencial orientar os cuidadores sobre os impactos do uso de telas para o desenvolvimento infantil e a importância do acompanhamento adequado.

## INTRODUÇÃO

O desenvolvimento infantil é marcado por influências normativas reguladas, com forte influência sócio-histórica, em que os acontecimentos significativos definem o comportamento e as atitudes de uma geração, como a presença categórica das inovações tecnológicas^(1)^.

Com o advento da tecnologia moderna, *smartphones, tablets*, computadores e brinquedos digitais foram incorporados à rotina das crianças que, por vezes, estão ocupadas com conteúdo digital. A utilização exagerada dessas tecnologias resulta em muitos desafios para a saúde, o desenvolvimento e o comportamento das crianças^(2,3)^.

Estudo identificou que 63,3% das crianças (n=180) entre 24 e 42 meses de idade são expostas a um tempo de telas digitais igual ou superior a duas horas/dia^(4)^, contrariando as recomendações brasileiras e internacionais de utilização de até uma hora de tela por dia na faixa etária de 2 a 5 anos^(5,6)^.

O tempo e a forma de uso de telas digitais estão relacionados aos benefícios e/ou malefícios dessa prática na vida das crianças. Entre as influências positivas, tem-se o aperfeiçoamento de habilidades de aprendizagem, o fácil acesso às chamadas de vídeo com familiares, o aprimoramento de sua criatividade e a autoexpressão. Por outro lado, de forma negativa, tem-se o vício em tecnologia, o aumento do nível de estresse, a redução de atividades físicas, os atrasos de linguagem, a falta de sono, o sofrimento emocional, e os problemas comportamentais e de relacionamento^(3)^.

Entre as crianças que estão expostas aos malefícios das telas digitais, por seu tempo de uso excessivo, estão aquelas que se encontram em situação de vulnerabilidade social. Estudo que avaliou 13.891 crianças europeias em vulnerabilidade social, caracterizadas pela ausência de rede de apoio social dos pais, composição familiar não tradicional, condição de migração e/ou desemprego de um ou ambos os responsáveis, identificou maior risco de elas apresentarem tempo de uso excessivo de telas digitais e de serem menos ativas em grupos esportivos, em comparação com grupos não vulneráveis^(7)^.

No Brasil, dados apontam que 32 milhões de crianças e adolescentes se encontram em situação de vulnerabilidade e de privação no país. Pesquisa liderada pelo Fundo das Nações Unidas para a Infância identificou que, entre os oito indicadores que compõem a pobreza multidimensional, três deles apresentaram piora expressiva entre 2020 e 2022, nomeadamente alimentação, educação e renda. Por outro lado, o acesso à informação, isto é, à TV e à internet em casa, apresentou uma melhora significativa até 2020, contudo ainda é muito desigual^(8)^.

O conceito de vulnerabilidade social pode ser aplicado a pessoas que vivenciam situações de adversidade, estando associado a fatores de risco que afetam negativamente as pessoas e o seu cotidiano. Esses fatores de risco, a exemplo do uso excessivo de telas digitais por crianças, podem ser condições ou comportamentos que provocam efeitos negativos ou indesejáveis e que aumentam a probabilidade de consequências adversas para o desenvolvimento psicossocial, com potencial para comprometer a saúde e o bem-estar do indivíduo^(9)^.

Os pais possuem um papel crucial na organização do uso das telas por seus filhos que, pela própria fase da vida, estão mais vulneráveis às adversidades das telas digitais. Configurações extras e senhas nos aparelhos eletrônicos, além de remoção das telas digitais dos quartos, estabelecimento de limites de tempo e vigilância do seu próprio tempo de telas, são estratégias viáveis que os pais podem adotar para a mitigação do uso exacerbado das telas digitais pelas crianças^(10)^.

Apesar da importância dos pais, é escasso o conhecimento deles sobre as recomendações de tempo e as formas de uso de telas digitais por crianças, bem como seus benefícios e malefícios. Pesquisa desenvolvida nos Estados Unidos, intitulada “*Common Sense Media*”, evidenciou que cerca de 60% dos pais de crianças de 0 a 8 anos possuem, de forma preponderante, compreensões de que suas crianças utilizam a quantidade certa de tempo de telas, e a maioria enfatiza não estar preocupada com as repercussões das telas sobre as crianças e a qualidade do conteúdo disponível para elas^(11)^.

A compreensão dos cuidadores, educadores e profissionais de saúde sobre os riscos potenciais da utilização excessiva de telas é um ponto fundamental para a promoção do desenvolvimento saudável das crianças, pois, a partir disso, outras ações que favorecem as habilidades cognitivas, linguísticas e socioemocionais são mais valorizadas e, consequentemente, inseridas nas rotinas das crianças^(10)^.

Assim, intervenções educativas precoces e apropriadas, destinadas ao grupo de cuidadores, constituem uma demanda de saúde pública e um compromisso com o desenvolvimento infantil global. Nesse contexto de saúde, o enfermeiro é um importante agente para promover diálogos que contemplem estímulos para o desenvolvimento infantil, redução de atividades passivas, conteúdo e tempo de uso das telas^(12)^. Isso é fundamental, visto que o ambiente familiar é o principal local onde as crianças utilizam tecnologias digitais. A mediação parental sobre o uso de tecnologia digital e o tempo de tela devem fazer parte das interações para pais/cuidadores e crianças. No entanto, a permissividade em relação ao acesso e uso excessivo da tela tem sua principal causa na necessidade parental em ocupar a criança^(13)^. Indo ao encontro da literatura, emerge a seguinte questão: quais são as percepções de cuidadores de pré-escolares de diferentes estratos de vulnerabilidade social quanto ao uso de telas por suas crianças?

## OBJETIVOS

Compreender as percepções de cuidadores sobre o uso de telas digitais por crianças de diferentes estratos de vulnerabilidade social.

## MÉTODOS

### Aspectos éticos

Este estudo corresponde à terceira fase de macroprojeto intitulado “Promoção da saúde de pré-escolares em vulnerabilidade social: um estudo de intervenção em creches de João Pessoa-PB”, aprovado pelo Comitê de Ética em Pesquisa de uma instituição pública de ensino superior da Paraíba.

A pesquisa contemplou as orientações éticas da Resolução n° 466/2012 do Conselho Nacional de Saúde. Os participantes foram esclarecidos acerca da voluntariedade em contribuir com o estudo, dos seus riscos e da assinatura do Termo de Consentimento Livre e Esclarecido. O anonimato foi mantido a partir da sigla “GF” (GF1, GF2... GF8), que consiste na ordem cronológica de realização dos grupos focais (GFs), somada à qualidade escolhida pelo cuidador na dinâmica inicial do GF e ao estrato de vulnerabilidade, como “GF5-Coragem, I”.

### Referencial teórico-metodológico

Trata-se de estudo desenvolvido sob a ótica das dimensões do uso de telas digitais propostas por Fitzpatrick *et al*., que são tempo de tela, conteúdo, natureza e contexto. Portanto, os dados foram analisados a partir do tempo de tela, que corresponde à duração da utilização das telas; a partir do conteúdo, se são vídeos e aplicativos educacionais, velocidade da ação apresentada nos vídeos e se adequados para a faixa etária, pró-sociais ou violentos; a partir da natureza, se a sua utilização se dá de forma passiva ou ativa; e a partir do contexto, que representa o momento, o local/ambiente físico e as justificativas dos cuidadores para utilizar as telas com suas crianças^(14)^.

### Tipo de estudo

Estudo qualitativo, descritivo e exploratório, com a utilização do *Consolidated criteria for Reporting Qualitative research* como guia para orientar o relatório do processo da pesquisa.

### Cenário do estudo

A pesquisa ocorreu em oito Centros Municipais de Educação Infantil (CMEIs), também denominados creches, de João Pessoa, Paraíba, Brasil. Em duas creches, não foi possível realizar os GFs por motivos de violência na comunidade e interdição para reforma.

Em 2022, o município contava com 93 CMEIs, que foram classificados em cinco estratos de vulnerabilidade social, a depender do bairro que estavam localizados (I - muito baixa vulnerabilidade; II - baixa vulnerabilidade; III - média vulnerabilidade; IV - alta vulnerabilidade; V - muito alta vulnerabilidade) e das variáveis renda, infraestrutura, educação, gênero, trabalho e composição familiar, conforme a topografia social do município^(15)^.

Tais variáveis são estabelecidas em Norma Operacional Básica/Sistema Único de Saúde, que apresenta as bases metodológicas para dimensionar a presença de vulnerabilidades sociais em um determinado território, de modo que a presença de uma única característica acima mencionada já indica vulnerabilidade social em algum grau, a saber: 1) renda (*per capita* familiar inferior a ¼ salário mínimo (SM); *per capita* familiar até ½ SM); 2) infraestrutura (domicílios inadequados; domicílios com dois a três moradores por dormitório (densidade 3:1)); 3) educação (pessoa de 4 a 14 anos que não frequenta escola; responsáveis com menos de quatro anos de estudo); 4) gênero (mulheres chefes, sem cônjuge); 5) trabalho (pessoa maior de 25 anos, desocupada, com quatro anos de estudo; pessoa de 10 a 15 anos que trabalha); 6) composição familiar (presença de pessoa de 60 anos e renda *per capita* familiar menor que ½ SM; presença de pessoa com deficiência e renda *per capita* familiar menor que ½ SM; número de crianças até 14 anos com renda *per capita* familiar até ½ SM e responsável com menos de quatro anos de estudo; número de filhos com menos de 15 anos; e mulher chefe, sem cônjuge)^(15)^. Cabe destacar que essa distribuição é consonante com a topografia social do município, disponível em 2022.

Nesse contexto, a seleção das creches se deu por amostragem estratificada proporcional, adotando-se procedimento aleatório a partir de dados disponibilizados pela Secretaria Municipal de Educação.

### Procedimentos metodológicos

A intervenção educativa sobre o uso de telas digitais foi desenvolvida com os cuidadores dos pré-escolares regularmente matriculados nas creches, a partir de rodas de conversa, vídeos e dinâmicas interativas (segunda fase do projeto). Ao final da intervenção educativa em cada creche, foi pactuada com o grupo de cuidadores e a direção a data da próxima fase do estudo (GFs), segundo a disponibilidade dos cuidadores e a programação da creche. Na semana do encontro, foi enviado previamente um lembrete via aplicativo de mensagens.

### Participantes do estudo

Utilizou-se a técnica de amostragem intencional, sendo convidados a participar do estudo todos os cuidadores que eram cuidadores de pré-escolares devidamente matriculados nas creches e com faixa etária de 2 a menores de 5 anos, que participaram previamente da intervenção educativa. Não foram incluídos no estudo os cuidadores que chegaram atrasados, após a discussão do encontro (GF).

### Coleta e organização dos dados

A terceira fase do projeto ocorreu no período de outubro de 2023 a junho de 2024, entre um e três meses após a intervenção educativa para promoção da saúde infantil, a fim de amenizar o viés de memória.

O GF foi a técnica de coleta de dados implementada, que tem como objetivo expressar opiniões que promovam o entendimento de comportamentos e percepções dos participantes^(16)^.

O GF foi conduzido em oito creches, sendo realizado um GF em cada creche. Foi conduzido por uma moderadora, a pesquisadora principal, apoiado por uma observadora, docente de instituição de ensino superior com experiência em GF, e por auxiliares (estudantes da graduação e pós-graduação) de um grupo de estudos em saúde da criança e do adolescente de uma universidade pública.

Para iniciar o GF, foi realizada a “dinâmica das qualidades”, que consistiu na leitura de uma lista de qualidades, e cada participante escolheu aquela que mais o representava. A qualidade escolhida foi escrita no crachá do participante, sendo o seu codinome “GF”.

Logo após, uma caixa surpresa continha palavras soltas, e os participantes foram convidados a formar uma frase, que consistia na questão disparadora do GF: “Como ocorre o uso de telas digitais pelo seu filho?”.

As discussões dos GFs foram audiogravadas e tiveram duração média de 60 minutos. O critério de encerramento adotado foi o de saturação, quando o moderador constata que o fenômeno pesquisado pode ser compreendido pelas informações já coletadas^(17)^. O *corpus* de dados foi transcrito na íntegra, porém não houve a validação dos participantes.

### Análise dos dados

Os dados empíricos foram analisados conforme a análise temática indutiva, uma técnica flexível e recursiva composta pelas seguintes fases: familiarização com os dados, transcrição dos discursos e leitura exaustiva do *corpus*; elaboração de códigos iniciais - codificação dos aspectos importantes e mais recorrentes e agrupamento dos extratos de dados referentes a cada código; busca por temas - junção dos extratos de códigos relacionados em temas em potencial; revisão dos temas - elaboração do mapa temático com seus subtemas e códigos e conferência de todos os extratos selecionados; definição e nomeação dos temas - ajustamento dos títulos dos temas, subtemas e códigos; produção do texto final - análise final dos extratos selecionados e da relação desses com a pergunta e objetivo da pesquisa^(18)^.

A análise do *corpus* de dados foi validada por duas pesquisadoras com experiência em pesquisa qualitativa (análise em pares). A pesquisadora principal realizou previamente a análise, validando-a, e, *a posteriori*, o material foi enviado para validação da coordenadora do projeto guarda-chuva, além de ter sido analisado pelo grupo de pesquisa.

Toda a análise do *corpus* de dados seguiu as recomendações nacionais sobre o uso de telas digitais por crianças, considerando o tempo de uso de até uma hora para crianças de 2 a 5 anos^(5)^; acima disso, o tempo foi considerado excessivo.

Para manter o anonimato dos participantes do GF, adotaram-se as siglas GF1, GF2 [...] GF8, que consistem na ordem cronológica de realização dos GFs, somadas à qualidade escolhida por eles na dinâmica inicial do GF (respeito, esperança, amor, entre outros) e ao estrato de vulnerabilidade social da creche (I, II, III, IV e V), resultando, por exemplo, no código “GF1-Gratidão, III” [...].

## RESULTADOS

Foram realizados oito GFs em creches dos estratos de vulnerabilidade social I, II, III e IV, sendo duas creches de cada estrato. Não foi possível realizar os GFs nas creches do estrato V por motivos de violência na comunidade e reforma do prédio.

Considerando as diferentes realidades sociais encontradas, foram realizados sete GFs no formato presencial e um remoto, o que favoreceu a coleta de dados com maior participação dos cuidadores. Assim, 30 cuidadores participaram dessa etapa, tendo, em média, quatro participantes em cada GF. A maioria era composta por mães das crianças, declaradas como principal responsável, com idade entre 18 e 30 anos, estado civil solteiro, 10 a 12 anos de escolaridade e com alguma atividade remunerada. As crianças, em sua maioria, estavam na faixa etária de 36 a 47 meses, eram do sexo feminino e raça parda.

A análise do *corpus* textual, que foi fundamentada nas dimensões relacionadas ao uso de telas, resultou na construção do tema “Elementos constituintes do uso de telas por crianças em vulnerabilidade social” e dos subtemas “Tempo de telas digitais”, “Contextos do uso das telas digitais”, “Natureza da utilização das telas digitais”, “Conteúdos consumidos nas telas digitais” e “Experiências dos familiares de pré-escolares com as telas digitais”.

### Tema: Elementos constituintes do uso de telas por crianças em vulnerabilidade social

#### Subtema I: Tempo de telas digitais

As telas digitais estão inseridas nos diferentes sistemas contextuais do desenvolvimento humano. Pensar sobre todos os aspectos, como tempo, contexto, conteúdo e natureza da utilização dessas por pré-escolares, permite uma compreensão ampliada de um fenômeno de grande impacto na primeira infância.

O tempo que as crianças empregam nas telas digitais é uma preocupação, pois todos os cuidadores relataram a utilização de telas por suas crianças, predominantemente, em torno de duas a três horas por dia, tempo considerado excessivo para a faixa etária.

*Olha, quando está comigo aqui, ela fica mais ou menos umas três horas, aí eu desligo. Por exemplo, na parte da manhã, ela acordou, aí vai brincar. Depois, eu deixo uma base de três horas só na TV, porque agora o tablet quebrou, então é só na TV.* (GF5-Coragem, I)

*Se eu deixar, ela passa umas cinco horas só na tela. Se não descarregar o telefone, se eu não tomar, ela fica.* (GF8-Respeito, III)

*Lá em casa, quando ele chega da aula, ele pede, e aí eu deixo umas duas horas. É o tempo que eu tenho para fazer minhas coisas. Ele fica de 18:00 às 20:00. Aí, quando dá mais ou menos 19:30, eu já vou desligando* [...]. *Eu desligo, e ele já não pede mais, não assiste mais. E de manhã, é esse intervalo, esse de meia horinha que ele vê.* (GF3-Amor, IV)

#### Subtema II: Contextos do uso das telas digitais

Os contextos de uso das telas digitais são diversos. As crianças estão nas creches nos turnos da manhã e tarde, o que as protege da exposição excessiva às telas no domicílio. Contudo, todo o seu tempo fora da instituição é permeado pelo uso de telas, muitas vezes para que o seu cuidador realize atividades domésticas, pessoais ou até mesmo no trajeto de casa até a creche.

*Não, eu colocava ele lá* [na televisão]*. Ele pedia para assistir, e eu deixava. Ia lavar minha loucinha, fazer o almoço. Para mim, eu sei que é errado, mas não tinha o que fazer. Deixava porque eu podia ajeitar as coisas, varrer a casa.* (GF1-Gratidão, III)

*É que eu tenho pouco tempo com eles porque eles são integral* [tempo na creche]*, então, assim, o meu tempo com eles é à noite. Aí eu tenho o meu curso, tenho o meu estágio, então o meu tempo é muito curto com eles. Então, mas é* [...] *infelizmente, as telas* [...]. (GF3-Respeito, IV)

*Na minha casa, o uso de telas não está muito legal, não. Até para vir* [para a creche], *tem que trazer o celular, senão ela vem chorando até aqui. Ela se habituou a ver o desenho dela.* (GF3-Paciência, IV)

*De manhã, ele também tem esse hábito. Na hora de estar vindo,* [para a creche] *no carro, aí ele sempre pede também o celular, e aí eu acabo dando, entendeu?* (GF3-Amor, IV)

No contexto de espaços públicos, como restaurantes e igrejas, as telas se tornaram uma “pseudoaliada” dos cuidadores para manter a criança em silêncio e quieta. Contudo, ressalta-se a decisão positiva de duas cuidadoras em não ofertar telas como “babá eletrônica” em espaços coletivos.

*Eu não vou mentir que, quando a gente sai, que eu quero que ele fique quieto. Eu digo: “Vai, mamãe, chegue, veja aqui”. Eu não vou mentir que eu faço. Eu sei que é errado, mas eu faço para ele ficar quieto, mas vou tentar me policiar para eu não fazer mais isso, porque está prejudicando ele.* (GF1-Gratidão, III)

*E, realmente, eu sou muito julgada e apontada, viu? E o menino não pode dar um choro que incomoda, se é no restaurante, incomoda. Porque você olha as crianças sentadas e estão tudo calada, mas estão tudo comendo, sem nem saber a cor da comida* [enquanto utilizam as telas]*. E o meu filho não, viu? Ele come sabendo o que ele come. Ele pede o que ele quer e ele come o que ele quer, assim, sabendo o que está comendo. Não está numa tela hipnotizada não, viu? Mas aí a gente é julgada.* (GF5-Resiliência, I)

*Eu fui para a missa com ele. Ele vê as outras crianças, e os pais dão* [a tela digital] *para poder assistir à missa. Aí ele veio pegar o meu. Eu disse: “Não, não pode. Aqui é a igreja, não pode usar celular”. Aí ele vem, vai, guarda na minha bolsa e se conforma* [...] *é bem conformado.* (GF8-Vitoriosa, III)

#### Subtema III: Natureza da utilização das telas digitais

O cuidador tem um importante papel na escolha dos contextos em que os seus filhos utilizarão as telas, assim como se o uso ocorrerá de forma passiva ou ativa (natureza). Os resultados apontaram a passividade das crianças diante das telas, ainda que alguns cuidadores tenham consciência da importância da sua presença para que as telas sejam um instrumento proveitoso para as crianças.

*É uma ferramenta, é uma faca de dois gumes, né? Porque, para ser uma ferramenta produtiva, tem que estar com o adulto acompanhando. E, muitas vezes, não dá para acompanhar. Isso é o grande processo da tela, porque tem muitas coisas. Meu filho não vê muito* [...] *ele vê curtas metragens, mas não vê muita bobagem. Vê vídeo de animais, tem muitas coisas bacanas e vídeo de experiência. Mas, para isso, tem que ser junto com os pais, e nunca dá para fazer esse uso.* (GF3-Gratidão, IV)

*Eu sempre estou muito perto. Se eu não estiver sentada com ele, se eu não estiver comigo na rede ou no chão, em algum canto, mas eu estou passando todo o tempo. Porque, para dar uma quebrada, né? Porque o menino fica “vidrado”, aí eu dou uma quebradinha.* (GF5-Resiliência, I)

A presença pontual dos cuidadores durante a utilização das telas pelas crianças se deu com o intuito principal de avaliação e modificação do conteúdo que já estava sendo consumido por elas.

*Quando eu vejo que está alguma coisa pesada, que eu escuto, eu vou lá e digo: “Não pode”, aí é na hora que ele faz as birras, sai e se joga lá no chão.* (GF1-Coragem, III)

*No meu caso, eles ficam sozinhos, mas aí eu fico regulando o que está assistindo, é claro. Quando eu escuto aquelas músicas do mundo, eu mando mudar, lógico* [...] *quando eu vejo, eu digo: “Muda isso aí, menina”, e ela muda. Do quarto, eu já escuto. Eu fico passando, às vezes, para regular, mas que eu fico 24 horas por perto, eu não fico, mas eu regulo. Que é o certo, não é? Mesmo que não esteja perto, tem que estar ali, olhando o que está fazendo.* (GF4-Amor, II)

#### Subtema IV: Conteúdos consumidos nas telas digitais

Os conteúdos que as crianças assistiam eram diversos, como vídeos disponíveis em plataformas digitais (*YouTube^®^, Kwai^®^* e *TikTok^®^*), músicas, desenhos infantis e filmes. Como o acesso às telas não era sistematicamente acompanhado pelos cuidadores, não se sabe se o conteúdo contemplava a classificação indicativa de conteúdos digitais para crianças.

*Chama de desenho, mas não é. Tem alguns* [vídeos] *que eu vejo e alguns que ela mesmo coloca no celular, no YouTube^®^. Quando eu vejo, eu coloco do meu jeito, mas, sem eu ver, ela que coloca. E ela com 3 anos, mais sabida do que eu.* (GF3-Paciência, IV)

*Porque eles assistem Kwai^®^, que não tem muita coisa boa, ensina algumas coisas que não prestam.* (GF4-Amor, II)

*Ela usava muito o TikTok^®^, que tem muitas coisas que são pesadas, aí eu tirei mesmo*. (GF2-Paciência, II)

*No momento que ele pede mais pelo desenho, por ele assistir pouco, eu deixo assistir, sabe?* [...] *às vezes, eu não coloco o desenho, coloco só as músicas pra ele ficar brincando, só escutando.* (GF5-Resiliência, I)

*Tem vezes que eu deixo a minha* [criança] *um tempinho a mais, mas tem vezes que ela pede para assistir filme comigo. Aí eu troco, porque ela está assistindo. Coloco um filme de criança, depois eu coloco um filme um pouquinho de adulto, mas não tem cenas mais assim, não.* (GF4-Gratidão, II)

No que diz respeito ao tipo de tela digital, a televisão foi compreendida pelos cuidadores como de escolha para uso no domicílio, por considerarem menos nociva à criança, em detrimento dos celulares e *tablets*.

*Não dou* [*tablet*]*, o máximo que ela assiste normal é na televisão, o programa mesmo normal. Eu tinha comprado um tablet para ela, para ela e a irmã dela, só que, como eu vi que os irmãos estavam demais, eu já recolhi o da irmã também. Aí o tablet está lá no guarda-roupa guardado há três meses, sem nenhuma das duas entrar, porque eu fui me estressando tanto com os adolescentes que eu vejo a hora elas embarcarem do mesmo jeito.* (GF1-Fortaleza, III)

*Eu não dou o celular para ela. Deixa mais a TV no YouTube^®^* [...] *bom, agora é somente a TV porque o tablet dela quebrou. O celular eu dou pouco, então é só a TV.* (GF5-Coragem, I)

*No celular, ele não pega, não, porque a gente não deixa. A avó deixa ele assistindo. Não quer brincar, nem nada. Ele até manda mudar o canal na televisão.* (GF4-Paciência, II)

#### Subtema V: Experiências dos familiares de pré-escolares com as telas digitais

A compreensão do uso de telas digitais pelos membros do núcleo familiar da criança é crucial, por ter impacto direto na relação que o pré-escolar tem com as telas. Conforme relatos dos participantes do estudo, o uso das mídias digitais, seja quanto ao tempo, contexto, conteúdo ou formas, também é comum aos familiares, de maneira livre e sem restrições.

*Lá em casa é bem dizer 24 horas. É televisão, o mais velho fica no tablet, jogando. E, assim, na TV, o que eu mais gosto de assistir é novela lá em casa. Estou assistindo novela, aí fica tudinho assistindo. Aí meu filho mais velho pelo menos gosta dessa novela; fica eu, ele e o pai. Já o pequeno* [a criança matriculada na creche] *fica no celular. Ele usa muito o YouTube^®^, que eu vou tentar tirar.* (GF1-Coragem, III)

*A minha menina, se eu tomar* [celular]*, ela chora demais. Não para um minuto, só para se der o celular na mão dela. Eu não sei o que fazer. Aí é dor de cabeça* [...] *se não der, briga. Lá em casa, são quatro crianças que ficam brigando pelo telefone.* (GF8-Respeito, III)

Ao falarem sobre o uso abusivo das telas digitais no microssistema por todo o núcleo familiar, alguns cuidadores suscitaram o sentimento de culpa e o desejo de mudança dessa prática.

*Eu me culpo muito porque eu fico bastante também no celular. Eu culpo meu marido, ele me culpa também, porque a gente fica bastante* [na tela] [...] *eu estou tentando me policiar também, tanto eu quanto meu marido, porque a gente sabe que é culpa nossa também esse comportamento dele. Isso me angustia demais, aí eu estou tentando me policiar para também não ficar tanto quanto eu ficava antes.* (GF1-Gratidão, III)

*Eu tenho que fazer o máximo para não mexer muito em celular.* (GF1-Fortaleza, III)

## DISCUSSÃO

A partir das percepções de cuidadores, apreendeu-se que as telas digitais estão imersas no microssistema das crianças e de suas famílias de diferentes estratos de vulnerabilidade social. Elas as utilizam por tempo excessivo, em contextos e com conteúdos diversos, de forma passiva. A prática do uso dos eletrônicos sem restrições no núcleo familiar foi revelada, resultando no sentimento de culpa dos cuidadores, porém com o desejo de mudanças.

A utilização de telas se tornou uma realidade nos lares das crianças no Brasil e no mundo, sendo motivo de preocupação com os impactos no desenvolvimento neuropsicomotor das crianças^(19)^. Os estímulos ofertados na primeira infância são essenciais para a evolução da criança. Dessa forma, as interações sensoriais (tato, olfato, estímulos proprioceptivos) devem vir das representações familiares e ambientais para promover condições positivas para o desenvolvimento da criança, não de representações fictícias^(20)^.

Os dados revelam que o tempo de uso das telas de pré-escolares se encontra em torno de duas a três horas por dia, conforme relatos dos cuidadores, sendo considerado excessivo quando se analisam as recomendações disponíveis sobre a temática^(5,6)^. Esse não é um achado isolado, pois estudo realizado com 180 crianças, de 24 a 42 meses, matriculadas em creches mostra que 63,3% contabilizaram tempo de tela superior a duas horas por dia^(4)^, podendo trazer impactos ao desenvolvimento da criança.

No que diz respeito ao desenvolvimento da linguagem, por exemplo, estudo realizado com crianças na faixa etária de 1 a 3 anos, que objetivou investigar a influência da quantidade, conteúdo e contexto das mídias digitais, evidenciou consequências negativas relacionadas ao contexto de telas no vocabulário de crianças de 12 a 16 meses. Aliado a isso, crianças de 17 a 36 meses também demonstraram efeitos negativos potencializados pelo maior tempo de exposição às mídias digitais^(21)^.

Estudos recentes apontam que o impacto do uso das telas na saúde e no desenvolvimento infantil varia significativamente de acordo com o conteúdo, o tempo de exposição e a interação social envolvida. Pesquisa com crianças chinesas de 3 a 6 anos identificou que programas não direcionados às crianças elevam o risco de problemas de saúde mental^(22)^. Outro estudo mostra que o tempo passivo em frente à tela (como assistir passivamente televisão) é preditor de menor qualidade de vida, principalmente no bem-estar físico e na aceitação social^(23)^.

Conforme as participantes do estudo, as crianças utilizam as telas digitais em contextos diversos, tanto em espaços públicos quanto em privados, principalmente no ambiente domiciliar. Os responsáveis apontaram a utilização das ferramentas digitais com a finalidade de manter as crianças quietas enquanto realizavam outras atividades domésticas, ou de outra natureza, em espaços coletivos. Contudo, alguns cuidadores tiveram entendimento diferente sobre o uso das telas em espaços públicos e, quando permitiram à criança ser criança, ou seja, quando elas não tinham acesso às telas, mas a brincadeiras com outros instrumentos ou brinquedos, sentiram-se julgados pelas pessoas. Além disso, estudo brasileiro que investigou a relação entre tempo de uso de tela em crianças de até 48 meses de idade e seus responsáveis evidenciou uma associação significativa entre o tempo de tela diário com uma maior prevalência de sintomas maternos de ansiedade e depressão^(24)^.

Pesquisas também demonstram que as telas digitais, seja no ambiente domiciliar, em outros ambientes privados ou em espaços públicos, são muito utilizadas como “babás digitais”, no intuito de auxiliar nas necessidades parentais e manter as crianças entretidas, quietas e ocupadas enquanto os pais podem desfrutar de momentos de descanso ou realizar atividades domésticas sem a interrupção das crianças^(12,25,26)^. Contudo, no ambiente familiar, o consumo das telas pode decorrer do frágil conhecimento parental sobre os impactos do uso no desenvolvimento social na primeira infância^(12)^.

A baixa escolaridade dos cuidadores e/ou a escassez de acesso à informação em saúde são relevantes fatores a serem considerados no uso de telas por crianças em vulnerabilidade, pois a vulnerabilidade social não está associada apenas à baixa renda e à instabilidade financeira, mas também a fragilidades em diferentes aspectos da vida, aos vínculos afetivo-relacionais e à desigualdade de acesso a bens e serviços públicos^(27)^.

Estudo desenvolvido na Suíça com adolescentes definiu a vulnerabilidade social a partir do *status* socioeconômico da família, relacionamento com os pais e desempenho acadêmico, e os classificou em não vulnerável, moderadamente vulnerável e altamente vulnerável. Os grupos de adolescentes moderadamente e altamente vulneráveis apresentaram risco maior de serem viciados em *smartphone* ou *internet*, além de a prevalência de tempo excessivo de tela ter aumentado com o grau de vulnerabilidade^(28)^.

A orientação aos pais quanto às telas digitais é eficaz para promover uma mudança nos comportamentos do tempo de telas das crianças. Ensaio clínico randomizado indiano, com cuidadores de crianças entre 2 e 5 anos, teve como foco o letramento dos pais sobre tempo de tela e o ambiente doméstico de mídia, constatando redução significativa no tempo médio de telas na avaliação pós-intervenção no grupo de intervenção, em comparação com o de controle^(29)^.

Outro fator que influencia positivamente o uso de telas por crianças é a sua presença regular em creches. Pesquisa norte-americana com 235 díades pais-filhos menores de 2 anos constatou que aqueles que participavam de creches assistiram a 43 minutos a menos de telas do que os que não participavam^(30)^.

Quando as telas estão sendo consumidas, é crucial que elas sejam visualizadas de forma compartilhada entre as crianças e seus responsáveis, pois é uma prática protetora que favorece a interação e o diálogo, reduzindo os impactos negativos das tecnologias no desenvolvimento infantil^(31)^ e os problemas relacionados à saúde e à segurança das crianças em ambiente online^(32)^. Entretanto, foi identificado que, por vezes, as crianças estão desacompanhadas, o que dá margem para o consumo de plataformas digitais e conteúdos inapropriados ao público infantil, conforme classificação indicativa.

As crianças não possuem maturidade nem discernimento suficientes para lidar com o conteúdo apresentado na *internet*, estando em risco sua segurança, privacidade e proteção social. Consequentemente, são submetidas ao risco de serem alvos de violências e abusos *online*, como nudes, *sexting* (envio de conteúdos eróticos), estupro virtual, *grooming* (aliciamento *online*), redes de pornografia, redes de pedofilia, trotes, desafios perigosos, *phishing* (obtenção de informações ilegalmente), *deepfakes* (alteração de vídeos ou fotos com ajuda de inteligência artificial), *cyberstalking* (assédio digital que envolve meios eletrônicos para perseguir a vítima) e *cyberbullying* (prática agressiva de intimidações e perseguições no ambiente virtual)^(32)^.

Diante de problemas tão sérios, a vigilância do conteúdo consumido pela criança não deve acontecer de forma pontual e com o intuito apenas de mudança do conteúdo. Das músicas e desenhos infantis às plataformas digitais como *Kwai^®^* e *TikTok^®^*, existe um mundo de possibilidades desfavoráveis à saúde da criança. Estudo ratificou que os vídeos em plataformas de mídia, como *TikTok^®^* e *Instagram^®^*, e desenhos animados são os principais conteúdos consumidos pelas crianças, mas que a maioria é imprópria para idade da criança^(33)^, conforme a Classificação Indicativa^(34)^.

No tocante ao uso da televisão pelas crianças, os cuidadores a consideram melhor para ser utilizada pela criança em detrimento de mídias interativas, como *smartphones, tablets* e *videogames*; por isso, priorizam o uso da televisão pelos seus filhos.

Estudo brasileiro com 180 crianças na faixa etária de 24 a 42 meses encontrou que, entre as 94,5% crianças que estavam expostas às telas, 61% utilizavam a televisão, 41%, *smartphones*, e 22%, *tablets*. Logo, a televisão ainda é a principal responsável pela exposição das crianças às telas^(4)^. Em contrapartida, outro estudo com 104 crianças evidenciou que 88,5% (n= 92) utilizam os *smartphones* para consumo do conteúdo digital, demonstrando que esse também representa um meio significativo para a exposição às mídias^(34)^.

Independentemente do tipo de tela, o seu uso será amplamente influenciado pela prática dos pais^(35)^, denominado de tecnoferência parental. Com o aumento do uso dos *smartphones* na rotina diária, a tecnoferência (interferência da tecnologia nos relacionamentos) surge como uma nova ameaça às relações familiares e ao desenvolvimento infantil. Estudo realizado com 826 pais identificou que 50% das crianças às vezes usavam o *smartphone* ao interagir com colegas, e 41%, ao interagir com os pais. Todavia, o uso dos *smartphones* durante atividades entre pais e filhos/em família, nas refeições ou viagens em família e em atividades pessoais das crianças, como estudar e dormir, é uma prática que tem gerado insatisfação entre os pais^(36)^.

No presente estudo, os cuidadores reconheceram o uso demasiado das telas pela criança e por todo o núcleo familiar. Ao se sentirem culpados por essa realidade, expressaram o desejo de mudança dessa prática. Ressalta-se que o despertar para a mudança do comportamento diante do uso das telas pode ser oriundo das orientações recebidas na intervenção educativa. Todavia, o sentimento de culpa pode ter limitado os seus relatos. Nesse contexto, pesquisa que avaliou o impacto da educação parental focada na limitação do tempo de tela na primeira infância demonstrou que, após intervenção por meio de grupo educacional, as crianças apresentaram mudança significativa no tempo de exposição às telas, com impacto positivo nas suas competências de desenvolvimento e comportamento^(37)^.

O tempo demasiado das telas digitais impede que as crianças vivenciem as interações dos ambientes domésticos^(38)^. Incluí-las nas atividades do ambiente doméstico, com sua participação adaptada nos afazeres domésticos, por exemplo, auxiliará na diminuição da utilização dos aparelhos eletrônicos, fortalecendo os vínculos e as interações entre pais e filhos.

Logo, é oportuno destacar que a educação em saúde para os cuidadores, especialmente os de alta vulnerabilidade social, é valiosa por permitir o acesso a orientações sobre as formas e os tempos de uso de telas por crianças, bem como a informações sobre os impactos de sua exposição excessiva. Ela também pode sugerir atividades que promovam o crescimento e o desenvolvimento saudáveis fora das telas digitais.

Considerando que o conhecimento dos cuidadores sobre o uso de telas digitais na primeira infância não é igualitário, apreendeu-se que muitos cuidadores desconheciam as recomendações sobre o tempo de uso de telas por crianças, bem como suas repercussões na saúde da criança. Foi, portanto, evidenciada a influência positiva após a atividade educativa, demonstrado pelo entendimento das mães sobre o impacto do uso de telas e desejo de mudanças em relação ao cenário de exposição.

Portanto, o uso excessivo, passivo e irrestrito de telas por crianças foi desvelado nos diferentes estratos de vulnerabilidade social, o que foi corroborado por outros estudos que afirmam esse desfecho desfavorável^(20-22)^. Além disso, algumas crianças poderão ter maiores prejuízos, devido às desigualdades sociais existentes nos grupos em contexto de maior vulnerabilidade social^(7)^, ratificando a necessidade de atenção à temática com foco na educação em saúde.

### Limitações do estudo

Entre as limitações, estão a não inclusão dos professores das creches - cuja relevância no cuidado às crianças e na educação dos cuidadores sobre o uso das telas digitais é inegável - e a estratificação social adotada, pois, apesar de serem dados oficiais do município, não classificam a vulnerabilidade familiar, mas a região em que a creche está localizada.

### Contribuições para as áreas da enfermagem e saúde

Ao apresentar resultados que capturam o contexto, a natureza e o conteúdo das telas, o estudo traz um olhar inovador e aprofundado das telas digitais quanto ao seu uso por crianças em vulnerabilidade social, contexto de extrema relevância para o cuidado em saúde e enfermagem, tendo em vista a necessidade de orientação aos pais e sociedade acerca dos efeitos das telas no desenvolvimento infantil e na saúde da população como um todo.

## CONSIDERAÇÕES FINAIS

Os cuidadores de crianças de diferentes estratos de vulnerabilidade social perceberam que o tempo de uso de telas digitais por seus filhos ocorreu, por vezes, de forma excessiva e em contexto diversos, com a finalidade de ocupar as crianças enquanto eles realizam suas atividades. Isso resulta em um uso passivo das telas pelas crianças, sem um acompanhamento adequado e limitado à avaliação esporádica do conteúdo consumido pela criança. Ademais, o uso da televisão é priorizado no domicílio, por ser percebida como uma tela menos nociva para a criança.

Sugere-se, portanto, a ampliação do acesso dos cuidadores às informações sobre as telas digitais e suas repercussões no desenvolvimento infantil, sobretudo daqueles que se encontram em maior vulnerabilidade social, bem como sobre as recomendações dos órgãos competentes. A percepção positiva dos cuidadores acerca das telas no desenvolvimento da criança ainda é uma realidade que pode contribuir para exposição excessiva e precoce das crianças às telas digitais, o que, a longo prazo, poderá resultar em uma geração dependente de tecnologias e impactar diferentes domínios do desenvolvimento infantil.

## Data Availability

Os dados de pesquisa estão disponíveis no corpo do artigo.
